# Clinical usefulness of the SAMe-TT2R2 score: A systematic review and simulation meta-analysis

**DOI:** 10.1371/journal.pone.0194208

**Published:** 2018-03-13

**Authors:** Jasper H. A. van Miert, Sarah Bos, Nic J. G. M. Veeger, Karina Meijer

**Affiliations:** 1 Department of Haematology, University Medical Center Groningen, Groningen, the Netherlands; 2 Department of Internal Medicine, University Medical Center Groningen, Groningen, the Netherlands; 3 Department of Clinical Epidemiology, University Medical Center Groningen, Groningen, the Netherlands; Inselspital Universitatsspital Bern, SWITZERLAND

## Abstract

**Background:**

Vitamin K antagonist (VKA) therapy is safer and more effective when patients have a high time within the therapeutic range and low international normalised ratio variability. The SAMe-TT_2_R_2_ score aims to identify those at risk for poor VKA control.

**Objectives:**

To evaluate the predictive value and clinical usefulness of the SAMe-TT_2_R_2_ score to identify those at risk for poor VKA control.

**Methods:**

We performed a systematic review in MEDLINE and Embase for original research papers assessing the SAMe-TT_2_R_2_’s relation to poor TTR. We performed a meta-analysis where scores ≥ 2 and ≥ 3 predicting TTR < 70%. When studies evaluated other cutoffs for TTR or SAMe-TT_2_R_2_, they were harmonised by multiple simulations with patient characteristics from the individual studies, if the data were available.

**Results:**

16 studies were identified and used in the meta-analysis: 4 and 2 times directly, 8 and 8 times harmonised for scores ≥ 2 and ≥ 3, respectively (not all studies provided information about both cutoffs). The sensitivities and specificities were too heterogeneous to pool. The positive likelihood ratios were 1.25 (1.14-1.38) for a score ≥ 2, and 1.24 (1.09-1.40) for a score ≥ 3; the negative ones were 0.87 (0.82-0.93) and 0.96 (0.91-1.02), respectively. This shows that the post-test probabilities hardly differ from the prior probability (prevalence).

**Conclusion:**

The SAMe-TT_2_R_2_ score does predict low TTR, but the effect is small. Its effect on individual patients is too limited to be clinically useful.

## 1 Introduction

Vitamin K antagonist (VKA) therapy is safer and more effective when patients have a high time within the therapeutic INR range (iTTR) [1] and low INR variability [2, 3]. However, the quality of anticoagulation achieved differs greatly between individuals. The first period of anticoagulant treatment provides some information about future quality [4], but it is unclear how long this “trial of VKA” should be. Ideally, one could identify patients prone to poor VKA control before starting treatment. Separate predictors have been identified before, but their combination was prognostically weak [5–7].

Apostolakis et al. developed a new tool to identify those prone to poor VKA control before starting treatment: the SAMe-TT_2_R_2_ score [8]. The score awards one point each for female sex; age <60 years; 2 or more of certain comorbidities; and the presence of interacting medication, and two points each for tobacco use and non-Caucasian race. The score was initially developed to identify “outliers” (i.e. those below a certain percentile of TTRs) [9, 10]. After further assessment in other studies, it evolved into proposed decision rules to give patients with a score of 2 or higher extra care [11], or suggest that those with a score >2 start a NOAC instead of trying VKA [12].

While it is not uncommon for a risk score’s area of use to expand, this could jeopardise the score’s validity. The aim of this tudy is to assess the predictive performance and added clinical benefit of the SAMe-TT_2_R_2_ score, using a systematic review and meta-analysis.

## 2 Methods

### 2.1 Selection criteria

Studies were required to meet all the following pre-defined inclusion criteria for the systematic review:

- Participants: patients on VKA, naive or experienced
- Test: SAMe-TT_2_R_2_ score
- Outcome: quality of anticoagulation (time in therapeutic range [13] or percentage of international normalised ratios in therapeutic range (PINRR); both henceforth called “TTR” for brevity)
- Type of study: published original research paper

The studies were required to provide data to derive or calculate test statistics (such as predictive values and likelihood ratios) from a 2x2 contingency table for inclusion in our meta-analysis.

### 2.2 Data sources and searches

We searched MEDLINE and Embase and included studies indexed up to 12 January 2017, the date of our last search. We used the search term *SAMe-TT2R2*, without limits on language or otherwise. We excluded MEDLINE citations in Embase. We checked references of the included studies.

### 2.3 Study selection

**Fig 1:**
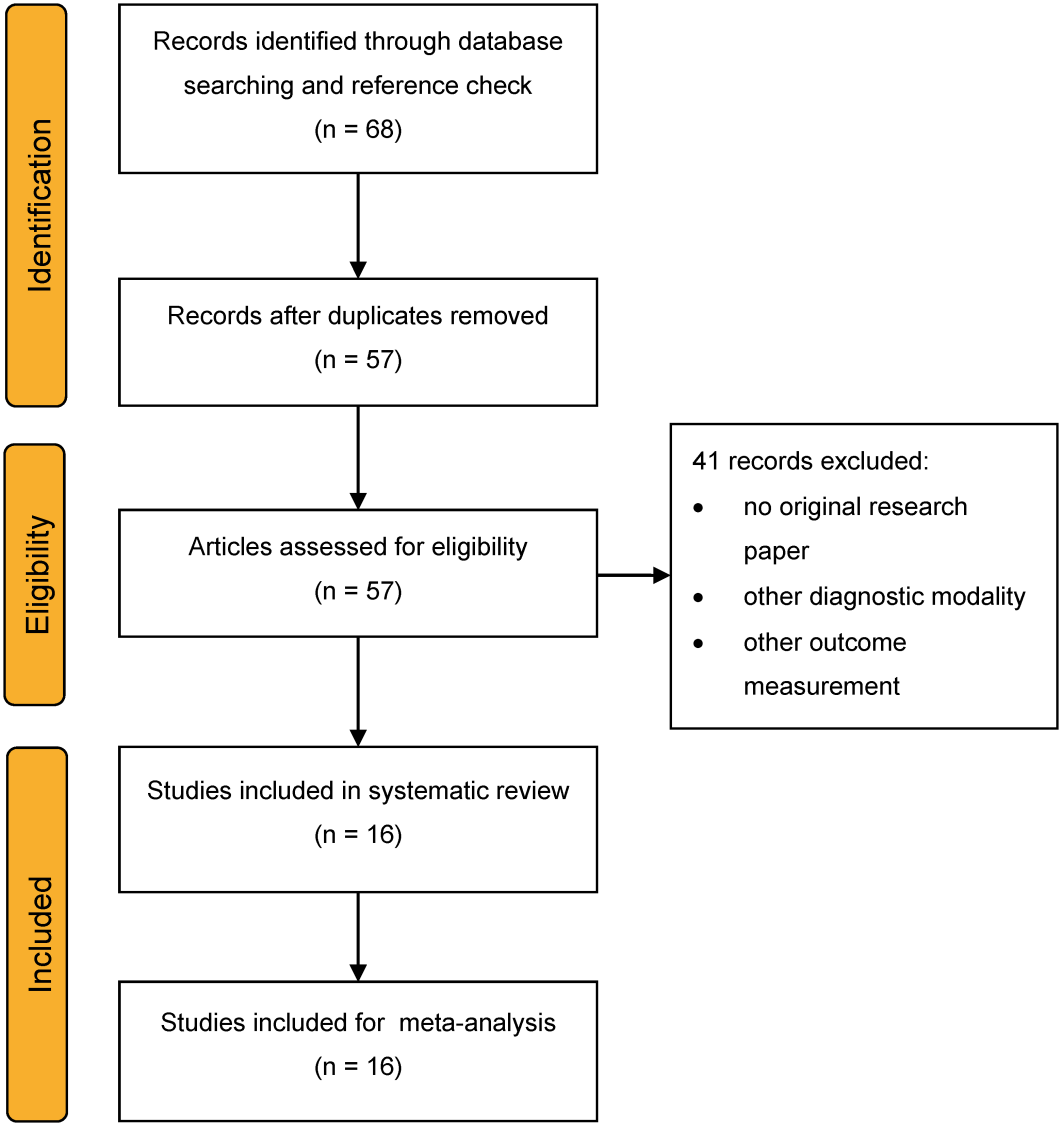
PRISMA flowchart [14] detailing the search strategy used.

Two independent reviewers (JvM and SB) performed the study selection individually based on the predefined inclusion and exclusion criteria. They screened all titles and abstracts of the articles to identify potentially eligible studies. The full text of these potentially eligible studies was then evaluated to determine eligibility for the systematic review and meta-analysis. Disagreements were resolved through discussion. There were no unresolved disagreements among the reviewers, which needed the advice of a third reviewer. When multiple studies were conducted on the same population of patients, we would extract data from the most complete publication or combine the results. The Preferred Reporting Items for Systematic Reviews and Meta-analyses (PRISMA) statement for reporting of systematic reviews and meta-analyses of randomised clinical trials was followed [14]. The PRISMA flowchart in Fig 1 shows the selection process; the PRISMA checklist is included in S1 Supporting Information. The study was not prospectively registered.

### 2.4 Data collection process

Two reviewers extracted data from each article independently (JvM and SB). Discrepancies between the reviewers were resolved by consensus. The following data were extracted from the included trials: indication for anticoagulation therapy, quality of anticoagulation achieved and its measurement method, numbers of patients, TTR cutoffs, SAMe-TT_2_R_2_ cutoffs, and test specifics. When the SAMe-TT_2_R_2_ cutoffs used in the study differed from those we chose, we modelled the different cutoffs if possible (see below).

### 2.5 Quality assessment

We rated the overall quality of evidence using the revised Tool for the Quality Assessment of Diagnostic Accuracy Studies (QUADAS-2 [15]; see S2 Table). Agreement on the quality of the individual studies was obtained after discussion (JvM and SB). If information to score a particular part of the assessment tool was absent we defined this risk of bias as unclear. Risk of bias of the index test was defined as unclear whenever SAMe-TT_2_R_2_ of 2 or 3 was not used as a cutoff to predict poor anticoagulation. We visually inspected funnel plots and performed a mixed-effects meta-regression model to assess possible publication bias.

### 2.6 Data synthesis

#### 2.6.1 Test statistics from original studies

We analysed SAMe-TT_2_R_2_ cutoffs of ≥2 and ≥3 (following from the aforementioned decision rules) to predict a TTR <70% (a TTR below the benchmark for high quality anticoagulation [11]). From articles that used the same TTR cutoff, we derived test statistics from the 2x2 contingency table (we algebraically calculated one based on information from the text when the contingency table was unavailable) with a spreadsheet tool [16].

#### 2.6.2 Harmonising cutoffs using a simulation

When a different TTR cutoff was used, we gathered the mean and standard deviation for each SAMe-TT_2_R_2_ category. This allowed us to simulate a TTR for every subject by sampling from a beta distribution set up to mimic a truncated normal distribution (because TTR is always between 0 and 100%). We created a 2x2 contingency table using cutoffs for TTR and SAMe-TT_2_R_2_, and used this to calculate test statistics. Every study was simulated thousand times, to incorporate the sampling uncertainty. These simulations were performed in R (R Foundation for Statistical Computing, Vienna, Austria) on Windows, using a script that is available as a supplement.

### 2.7 Data analysis

To assess the performance of our simulation, we simulated all studies with their original cutoff values, and compared the simulated test statistics with those originally found in the article.

We presumed heterogeneity in studies as a result of variation in VKA control achieved in different settings by different clinics, and indication for treatment. We pooled data using a random effects model, unless the outcomes were too heterogeneous in effect sizes (based on the forest plots) or had a too large I^2^. Likelihood ratios, negative and positive predictive values, sensitivity, specificity, and power of separation (difference between the post-test probabilities of the two groups [17]) are reported.

The meta-analysis was performed in R using the metafor package [18]. We report data as point estimate (95% confidence or reference interval) unless otherwise indicated.

## 3 Results

### 3.1 Study selection

We identified 57 distinct articles. We excluded 41 records, so 16 studies [8, 19–33] could be included in the systematic review and meta-analysis (see S1 Supporting Information).

### 3.2 Study characteristics

14 studies [8, 19–21, 23–26, 28–33] were performed in patients with atrial fibrillation; 2 [22, 27] were done in patients with venous thromboembolism. 5 studies [19, 22, 27, 29, 31] reported on VKA naive patients; 5 [23–25, 32, 33] on experienced patients and for 6 [8, 20, 21, 26, 28, 30] this was not reported. Study characteristics are summarised in Table 1.

**Table 1:** Study characteristics.

Study	Score	TTR	Ind	N	Cohort	Period excluded	TTR duration	TTR method
Abumuaileq [19]	≥ 2	< 70	AF	911	inception	first month	12 months or until event	PINRR
Abumuaileq [19]	≥ 2	< 65	AF	911	inception	first month	12 months or until event	PINRR
Apostolakis [8]	–	–	AF	286	not reported	not reported	not reported	Rosendaal
Bernaitis [20]	–	–	AF	1137	not reported	not reported	not reported	Rosendaal
Chan [21]	> 2	> 70	AF	1428	not reported	first 6 weeks	not reported	Rosendaal
Chan [21]	> 3	> 70	AF	1428	not reported	first 6 weeks	not reported	Rosendaal
Demelo [22]	≥ 2	< 65	VTE	135	inception	first month	not reported	Rosendaal
Gallego [23]	–	–	AF	972	experienced	none	6 months	Rosendaal
Gorzelak [24]	–	–	AF	104	experienced	none	6 months back	Rosendaal
Lip [25]	–	–	AF	229	experienced	not reported	not reported	Rosendaal
Lobos [26]	≥ 2	< 65	AF	1524	not reported	not reported	12 months back	Rosendaal
Lobos [26]	≥ 2	< 70	AF	1524	not reported	not reported	12 months back	Rosendaal
Lobos [26]	≥ 3	< 65	AF	1524	not reported	not reported	12 months back	Rosendaal
Palareti [27]	≥ 2	< 65	VTE	1308	inception	not reported	not reported	Rosendaal
Park [28]	–	–	AF	380	not reported	first month	not reported	Rosendaal
Poli [29]	–	–	AF	1089	inception	none	not reported	Rosendaal
Proietti [30]	> 2	< 70	AF	3624	mixed	mixed	not reported	Rosendaal
Proietti [30]	> 2	< 65	AF	3624	mixed	mixed	not reported	Rosendaal
Roldan [31]	≥ 2	< 65	AF	459	inception	not reported	6 months	Rosendaal
Ruiz [32]	< 2	> 65	AF	1056	experienced	not reported	6 months back	Rosendaal
Ruiz [32]	< 2	> 70	AF	1056	experienced	not reported	6 months back	Rosendaal
Szymanski [33]	–	–	AF	211	experienced	not reported	not reported	Rosendaal

AF: Atrial fibrillation; Ind: indication; N: number of patients included; period excluded: period excluded in calculation of the TTR; Score: SAMe-TT_2_R_2_ score; TTR: time in therapeutic range (here also percentage of INR’s in therapeutic range); VTE: venous thromboembolism

16 different studies were included in the meta-analysis: 12 for a SAMe-TT_2_R_2_ cutoff of ≥ 2, 10 for a cutoff ≥ 3. 8 and 8 studies were simulated before inclusion, respectively.

### 3.3 Quality assessment

The risk of systematic bias within studies was low. However, the specific methodology of many studies was unclear. Some studies provided insufficient data on patient selection; many studies did not provide enough information about the timing of the calculation of the SAMe-TT_2_R_2_ score and quality of anticoagulation. This could introduce survival bias: patients with poor VKA control may cease treatment. Multiple studies did not evaluate a cutoff for the SAMe-TT_2_R_2_ score or the TTR, but chose to evaluate the variables continuously. The quality assessment is summarised in S1 Table.

Due to the limited number of studies for each combination of score and TTR cutoffs, we could not assess publication bias for every combination. For those combinations where it was possible, we found no evidence for publication bias.

### 3.4 Results of individual studies

The articles used myriad ways to evaluate the SAMe-TT_2_R_2_ score: some authors used a high score to predict low TTR [8, 19, 22, 26, 27, 30–32], others a low score to predict high TTR [21, 32]. This affects the sensitivity and specificity. The cutoffs used to define a “high” score or a “low” TTR varied as well. Some studies evaluated multiple cutoffs for quality of anticoagulation or SAMe-TT_2_R_2_ [19, 21, 26, 30, 32]. The SAMe-TT_2_R_2_ cutoff ≥ 2 in combination with a TTR cutoff of 65 was studied most often: in 6 studies [19, 22, 26, 27, 31, 32] including 5393 patients. 6 studies [8, 20, 23–25, 29] were performed without cutoffs for SAMe-TT_2_R_2_ score or TTR, including 3817 patients. The results of the individual studies (recalculated to have a SAMe-TT_2_R_2_ ≥ cutoff predict a TTR < cutoff) are summarised in S2 Table.

The prevalence of TTR below the cutoff was 39–89%. The prevalence of a SAMe-TT_2_R_2_ score above the cutoff was 21–46% and 5–82% in studies that evaluated cutoffs ≥ 2 and 3, respectively. Sensitivity and specificity ranged from 6–82% and 14–96%, respectively. 4 studies [21, 22, 28, 33] showed that a high score made poor anticoagulation less likely (LR+ < 1).

There were no patients with a SAMe-TT_2_R_2_ score < 2 in three Asian studies [20, 21, 28], because the SAMe-TT_2_R_2_ score awards two points for non-Caucasian race. Another study’s [30] results could not be used for the simulation, so only the original cutoff could be used. Therefore, these studies could only be used to assess the score’s performance with a cutoff ≥ 3. Other studies only reported dichotomised SAMe-TT_2_R_2_ scores with a cutoff of 2 [22, 24–26, 31, 33]. These studies were excluded for the evaluation of the cutoff ≥ 3. From the study that introduced the SAMe-TT_2_R_2_ score [8], we only used the external validation cohort.

### 3.5 Validation of the simulation

**Fig 2:**
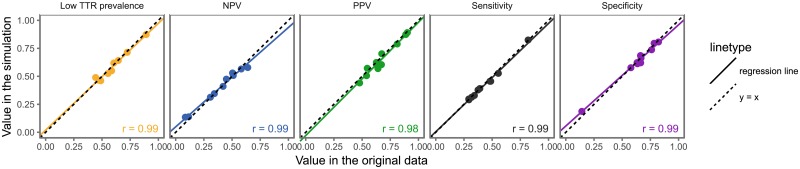
Calibration plot comparing simulated values with the corresponding values from the original studies.

We simulated all studies with their original cutoff values and compared the simulated point estimates and boundaries of the reference interval with their counterparts found in the studies. We did this for sensitivity, specificity, positive and negative predictive values, and prevalence of low TTR. This is graphically shown in Fig 2. Pearson’s correlation was 99%. The simulated point estimate fell in the original confidence interval in 82% of cases, and the differences between the original and simulated point estimates were small: mean < 0.01, SD = 0.03 (see also S1 Fig).

### 3.6 Meta-analysis

**Fig 3:**
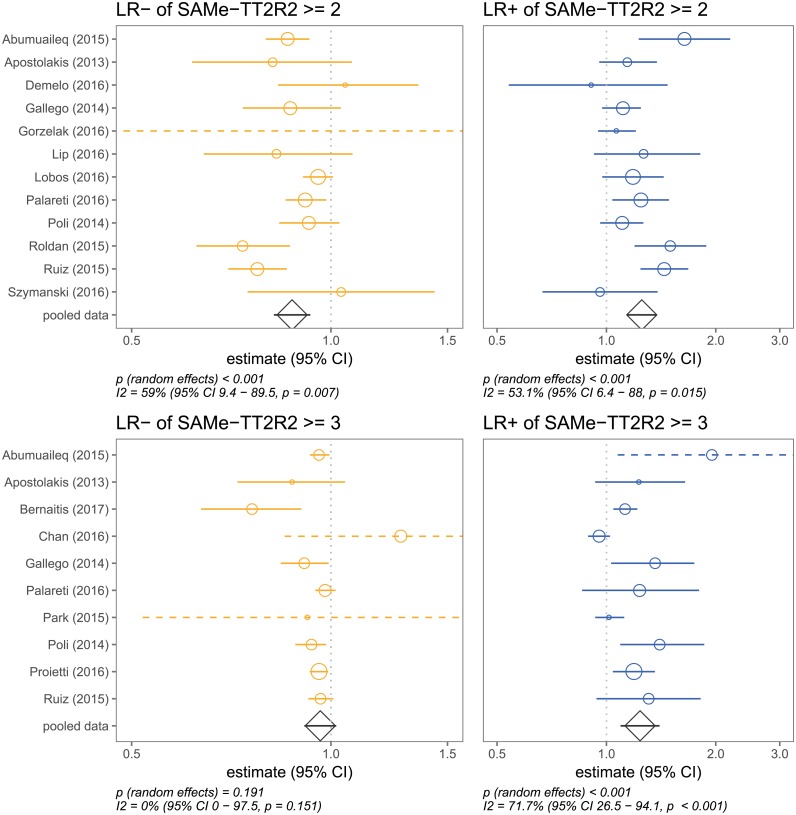
Forest plots showing positive and negative likelihood ratios (LR+, LR-) of the SAMe-TT_2_R_2_ score, using cutoffs of ≥2 and ≥3 to predict a TTR <70%.

The results of the meta-analysis are summarised in Table 2, Fig 3 and S2 Fig. We decided not to pool the data for sensitivities and specificities, because they were too heterogeneous (see Fig 3; lower bound of 95% CI of I^2^ >97%).

**Table 2:** Performance of the SAMe-TT_2_R_2_ score to predict TTR <70%.

SAMe-TT_2_R_2_	LR-	LR+	PSEP
≥ 2	0.87 (0.82–0.93)	1.25 (1.14–1.38)	0.08 (0.05–0.11)
≥ 3	0.96 (0.91–1.02)	1.24 (1.09–1.40)	0.06 (0.02–0.10)

LR-, LR+: negative and positive likelihood ratio, respectively; PSEP: power of separation; TTR: time in therapeutic range

## 4 Discussion

Vitamin K antagonist (VKA) therapy is safer and more effective when patients have a high time within the therapeutic INR range (iTTR) [1] and low INR variability [2, 3]. The SAMe-TT_2_R_2_ score [8] was developed to identify VKA control outliers before they started treatment. While the score has been adopted in AF guidelines [1], the added benefit of this score remains unclear. We evaluated how well the score identified those with a poor TTR (< 70%, which is below the European Society of Cardiology’s cutoff for high-quality anticoagulation [11]) with cutoffs from proposed decision rules [11, 12], using a systematic review and meta-analysis.

There is a striking difference in how studies applied and validated the SAMe-TT_2_R_2_ score. This process, from identifying those with poorest VKA control [8, 10] to evaluating the relationship with continuous [19, 20, 23] or categorised TTR values [21, 27, 28, 31, 32], fits the exploration of the score’s usefulness for individual patient care. This heterogeneity is however confusing, which is why we harmonised the different cutoffs. We evaluated SAMe-TT_2_R_2_ cutoffs of ≥ 2 (“patients who might need extra care” [11]) and ≥ 3 (“should start a direct oral anticoagulation instead of VKA” [12]).

**Fig 4:**
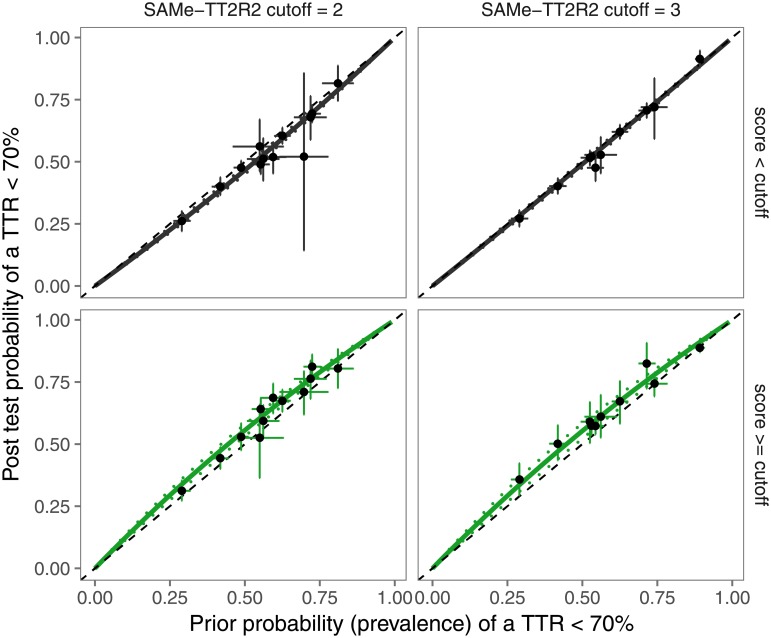
Pre-test and post-test probabilities plot for the possible SAMe-TT_2_R_2_ scores. Results from individual studies are indicated by dots, with the horizontal and vertical lines representing the 95% confidence interval.

The score’s sensitivity and specificity to identify a TTR <70% differed substantially between studies. A more consistent finding was that a test outcome does not decrease the uncertainty about VKA control substantially: the prior and posterior probabilities hardly differ (0.08 (0.05–0.11) and 0.06 (0.02–0.10) for cutoffs 2 and 3, respectively). This is also reflected in the likelihood ratios (LR+ 1.25 (1.14–1.38) and 1.24 (1.09–1.40); LR- 0.87 (0.82–0.93) and 0.96 (0.91–1.02)), which are very close to unity and graphically shown in Fig 4.

More important for clinical practice is whether a test manages to make the post-test probability surpass a clinical probability threshold: from a “grey area” of clinical uncertainty, to the certainty treatment is (un)necessary. It is unlikely that the SAMe-TT_2_R_2_ score is able to do this: the change in probabilities is too small. The pre-test probability of a poor TTR varies from setting to setting (e.g. by country, or with manual versus computer-assisted dosing). An estimate of this probability can be based on the TTRs achieved by other patients managed in a particular setting. In the Netherlands patients are managed by dedicated thrombosis services that publish statistics on the TTRs of their patients in their annual reports.

The other way around, one could ask the question in which populations the score could change clinical decision making. This depends on the clinical probability thresholds used. Imagine one wants to be 70% certain of poor VKA control before withholding VKA therapy, and will definitely start VKA therapy if the probability of poor TTR is less than 20%. A score ≥ 2 is only useful when the prior probability is between 65.1% (lowest prior probability which will result in a post probability ≥ 70%) and 69.9% (if the prior probability already equals the threshold, we do not need additional information). Likewise, a score < 2 is only useful for prior probabilities 20.1–22.2%. For a score cutoff of 3, these numbers are 65.3–69.9% and 20.1–20.6%, respectively. This underlines the limited clinical usefulness from the score.

Others have tried to predict an individual’s TTR. Rose et al. developed a more extensive prediction model, but its explained variation was low (3.2–6.8%) [5]. The same is true for the work of MacEdo et al. (7% variation explained) [6]. Mueller et al. [7] did not report the variance in TTR explained by the HAS-BLED score, but we estimated it with a simulation to be around 12%. Even pharmacogenetics-based warfarin dosing only moderately improved TTR [34]. This shows that there is a large unexplained inter-individual difference in the response to VKA.

### 4.1 Strengths and limitations

Our study has strengths and limitations. The studies we identified were heterogeneous in many aspects: the cutoffs used for the SAMe-TT_2_R_2_ score and TTR, the method to determine quality of anticoagulation, and the indication for anticoagulation therapy.

We used a simulation method to uniform the cutoffs and calculate their outcomes. This is a not yet established method, but we have shown this works very well. It allowed us to meta-analyse the results with established methods.

There was one study that did not report the TTR with the Rosendaal method, but instead counted the number of INR measurements within range. The two methods are not equivalent [35]. Sensitivity analysis showed the results did not change meaningfully when only studies using the Rosendaal method were included (see S3 Fig and S3 Table).

There was no difference in the score’s performance in patients with atrial fibrillation, compared with those with venous thromboembolism (S3 Fig and S3 Table). The assumption that the SAMe-TT_2_R_2_ score performs best in populations with a high probability of a low TTR and a large spread in TTRs could not be substantiated in post-hoc sensitivity analyses (S3 Fig and S3 Table).

Many studies conclude that the SAMe-TT_2_R_2_ score performs well based on a statistically significant C statistic or statistically significant differences in mean TTR between SAMe-TT_2_R_2_ groups. To answer our question, we evaluated different outcomes. Post-test probabilities of certain cutoffs (in this case SAMe-TT_2_R_2_ score ≥2 and ≥3) are relevant for clinical decision making. The C statistic summarises the performance of all possible cutoffs, and is more appropriate when no cutoffs have been defined. Furthermore, it assesses the probability of a certain test outcome given the presence or absence of disease, instead of the probability of poor TTR given a certain SAMe-TT_2_R_2_. A different mean TTR in SAMe-TT_2_R_2_ groups does not address the score’s discriminatory performance; there may be considerable overlap.

### 4.2 Conclusion

The SAMe-TT_2_R_2_ score does predict low TTR, but the effect is small. Its effect on individual patients is too limited to be clinically useful. Therefore, the evidence does not support the use of the aforementioned decision rules.

## Supporting information

S1 Supporting Information PRISMA flowchart. (PDF)

S1 Table Quality assessment of studies. QUADAS-2 rating.
(PDF)

S2 Table Results from individual studies. (PDF)

S3 Table Sensitivity analyses. Shows how different indications or methods of TTR measurement change the results.
(PDF)

S1 Fig Bland Altman plot. Shows the difference between the simulated and original values.
(PDF)

S2 Fig Forest plots showing sensitivity, specificity and power of separation (PSEP) of the SAMe-TT2R2 score. Uses cutoffs of ≥2 and ≥3 to predict a TTR <70%. PSEP: power of separation; TTR: time in therapeutic range.
(PDF)

S3 Fig Sensitivity analyses. Shows how different indications or methods of TTR measurement change the results. Dotted lines represent confidence intervals that were too wide to be displayed properly.
(PDF)
