# Supplementary material for: Clinical usefulness of the SAMe-TT2R2 score: A systematic review and simulation meta-analysis
Source: PLoS One. 2018 Mar 13;13(3):e0194208. doi: 10.1371/journal.pone.0194208 (PMC5849337; doi:10.1371/journal.pone.0194208)
Supplement: S1 Table — QUADAS-2 rating. (PDF) [file pone.0194208.s002.pdf]

Clinical usefulness of the SAMe-TT_2R_2 score

A systematic review and simulation meta-analysis

J.H.A. van Miert, S. Bos, N.J.G.M. Veeger, K. Meijer

S1 Table: Quality assessment of studies.

STUDY	RISK OF BIAS				APPLICABILITY CONCERNS		
	PATIENT SELECTION	INDEX TEST	REFERENCE STANDARD	FLOW AND TIMING	PATIENT SELECTION	INDEX TEST	REFERENCE STANDARD
**Abumuaileq [19]**	High	Unclear	Unclear	Low	Low	Low	Unclear
**Apostolakis [8]**	High	N/A	Unclear	Unclear	Unclear	N/A	Low
**Bernaitis [20]**	Unclear	Unclear	Unclear	Unclear	Low	Low	Unclear
**Chan [21]**	Unclear	Low	Unclear	Unclear	Unclear	Low	Low
**Demelo [22]**	Low	Unclear	Low	Low	Low	Low	Low
**Gallego [23]**	High	Unclear	Low	Unclear	Unclear	Low	High
**Gorzelak [24]**	Unclear	Unclear	Unclear	Unclear	Low	Low	Low
**Lip [25]**	Unclear	Unclear	High	Unclear	Low	High	High
**Lobos [26]**	Low	Unclear	Unclear	Unclear	Low	Low	Low
**Palareti [27]**	Low	Unclear	Unclear	Unclear	Low	Low	Low
**Park [28]**	Low	Unclear	Unclear	Unclear	Unclear	Low	Low
**Poli [29]**	Low	Unclear	Unclear	Unclear	Unclear	Low	Low
**Proietti [30]**	High	Unclear	Unclear	Unclear	Unclear	High	High
**Roldan [31]**	Low	Low	Low	Low	Low	Low	Low
**Ruiz [32]**	Unclear	Unclear	Unclear	Unclear	Low	Low	Unclear
**Szymanski [33]**	Unclear	Unclear	Unclear	Unclear	Unclear	Low	Unclear

According to the QUADAS-2. [15]
