# Supplementary material for: Clinical usefulness of the SAMe-TT2R2 score: A systematic review and simulation meta-analysis
Source: PLoS One. 2018 Mar 13;13(3):e0194208. doi: 10.1371/journal.pone.0194208 (PMC5849337; doi:10.1371/journal.pone.0194208)
Supplement: S2 Table — (PDF) [file pone.0194208.s003.pdf]

Clinical usefulness of the SAMe-TT$_2$R$_2$ score

A systematic review and simulation meta-analysis

J.H.A. van Miert, S. Bos, N.J.G.M. Veeger, K. Meijer

S2 Table: Results from individual studies.

Study	SAMe-TT2R2	TTR	Low TTR prev.	LR-	LR+	NPV	PPV	PSEP	Sensitivity	Specificity
Abumuaileq [19]	≥2	<65	0.60 (0.57-0.64)	0.90 (0.84-0.97)	1.35 (1.07-1.71)	0.42 (0.38-0.46)	0.67 (0.61-0.73)	0.09 (0.02-0.16)	0.29 (0.25-0.33)	0.78 (0.74-0.83)
Abumuaileq [19]	≥2	<70	0.72 (0.70-0.75)	0.86 (0.80-0.93)	1.64 (1.23-2.19)	0.31 (0.27-0.34)	0.81 (0.76-0.86)	0.12 (0.06-0.18)	0.29 (0.26-0.33)	0.82 (0.77-0.87)
Chan [21]	>2	<70	0.89 (0.88-0.91)	1.27 (0.85-1.91)	0.95 (0.89-1.02)	0.09 (0.05-0.12)	0.89 (0.87-0.91)	-0.03 (-0.06-0.01)	0.82 (0.80-0.84)	0.14 (0.09-0.20)
Chan [21]	>3	<70	0.89 (0.88-0.91)	0.95 (0.84-1.07)	1.11 (0.88-1.40)	0.11 (0.09-0.13)	0.90 (0.88-0.93)	0.01 (-0.02-0.05)	0.37 (0.35-0.40)	0.66 (0.59-0.74)
Demelo [22]	≥2	<65	0.49 (0.40-0.57)	1.02 (0.81-1.29)	0.95 (0.59-1.55)	0.51 (0.40-0.61)	0.48 (0.33-0.62)	-0.02 (-0.20-0.16)	0.32 (0.21-0.43)	0.67 (0.56-0.78)
Lobos [26]	≥2	<65	0.39 (0.37-0.42)	0.94 (0.89-1.00)	1.24 (1.02-1.50)	0.62 (0.59-0.65)	0.44 (0.39-0.50)	0.07 (0.00-0.13)	0.24 (0.20-0.27)	0.81 (0.78-0.83)
Lobos [26]	≥2	<70	0.49 (0.46-0.51)	0.96 (0.91-1.01)	1.18 (0.97-1.44)	0.52 (0.50-0.55)	0.53 (0.47-0.58)	0.05 (-0.01-0.11)	0.23 (0.20-0.26)	0.81 (0.78-0.83)
Lobos [26]	≥3	<65	0.39 (0.37-0.42)	0.98 (0.96-1.01)	1.39 (0.89-2.16)	0.61 (0.59-0.64)	0.47 (0.36-0.59)	0.08 (-0.03-0.20)	0.06 (0.04-0.08)	0.96 (0.94-0.97)
Palareti [27]	≥2	<65	0.59 (0.56-0.61)	0.86 (0.80-0.92)	1.44 (1.20-1.72)	0.45 (0.42-0.48)	0.67 (0.62-0.72)	0.12 (0.06-0.18)	0.34 (0.31-0.38)	0.76 (0.73-0.80)
Park [28]	≥4	<65	0.64 (0.60-0.69)	1.05 (0.88-1.25)	0.93 (0.72-1.19)	0.34 (0.28-0.41)	0.63 (0.55-0.70)	-0.03 (-0.13-0.07)	0.39 (0.33-0.45)	0.58 (0.49-0.66)
Proietti [30]	>2	<65	0.42 (0.41-0.44)	0.96 (0.93-0.99)	1.18 (1.03-1.34)	0.59 (0.57-0.60)	0.46 (0.43-0.50)	0.05 (0.01-0.09)	0.21 (0.19-0.23)	0.82 (0.80-0.83)
Proietti [30]	>2	<70	0.53 (0.51-0.55)	0.96 (0.93-0.99)	1.19 (1.04-1.36)	0.48 (0.46-0.50)	0.57 (0.54-0.61)	0.05 (0.01-0.09)	0.21 (0.19-0.23)	0.82 (0.80-0.84)
Roldan [31]	≥2	<65	0.44 (0.40-0.49)	0.71 (0.59-0.85)	1.50 (1.23-1.84)	0.64 (0.58-0.70)	0.55 (0.48-0.61)	0.18 (0.09-0.27)	0.55 (0.49-0.62)	0.63 (0.57-0.69)
Ruiz [32]	≥2	<65	0.47 (0.44-0.50)	0.82 (0.73-0.91)	1.32 (1.15-1.53)	0.58 (0.54-0.62)	0.54 (0.49-0.59)	0.12 (0.06-0.18)	0.48 (0.44-0.53)	0.64 (0.60-0.68)
Ruiz [32]	≥2	<70	0.55 (0.52-0.58)	0.77 (0.70-0.86)	1.44 (1.24-1.68)	0.51 (0.47-0.55)	0.64 (0.60-0.69)	0.15 (0.09-0.21)	0.49 (0.45-0.53)	0.66 (0.62-0.71)
Szymanski [33]	≥2	<70	0.81 (0.76-0.86)	1.04 (0.75-1.43)	0.96 (0.67-1.38)	0.18 (0.11-0.26)	0.80 (0.73-0.88)	-0.01 (-0.12-0.09)	0.46 (0.38-0.53)	0.52 (0.37-0.68)

LR-, LR+: negative and positive likelihood ratio, respectively; prev: prevalence; NPV: negative predictive value; PPV: positive predictive value; PSEP: power of separation; TTR: time in therapeutic range (here also percentage of INR's in therapeutic range)
