# Supplementary material for: Clinical usefulness of the SAMe-TT2R2 score: A systematic review and simulation meta-analysis
Source: PLoS One. 2018 Mar 13;13(3):e0194208. doi: 10.1371/journal.pone.0194208 (PMC5849337; doi:10.1371/journal.pone.0194208)
Supplement: S3 Table — Shows how different indications or methods of TTR measurement change the results. (PDF) [file pone.0194208.s004.pdf]

Clinical usefulness of the SAMe-TT_2R_2 score

A systematic review and simulation meta-analysis

J.H.A. van Miert, S. Bos, N.J.G.M. Veeger, K. Meijer

S3 Table: Sensitivity analyses.

SAMe-TT_2R_2	Sensitivity analysis		N	LR-	LR+	PSEP
≥ 2	Full analysis		12	0.87 (0.82-0.93)	1.25 (1.14-1.38)	0.08 (0.05-0.11)
	Indication	AF	10	0.86 (0.80-0.93)	1.26 (1.13-1.41)	0.08 (0.05-0.12)
		VTE	2	0.93 (0.84-1.02)	1.21 (0.92-1.58)	0.06 (-0.02-0.14)
	TTR method	PINRR	1	0.86 (0.80-0.93)	1.64 (1.23-2.19)	0.12 (0.06-0.18)
		Rosendaal	11	0.88 (0.81-0.94)	1.21 (1.10-1.33)	0.08 (0.04-0.11)
	Origin	original data	4	0.88 (0.79-0.99)	1.34 (1.10-1.64)	0.09 (0.02-0.16)
		simulation	8	0.87 (0.80-0.94)	1.18 (1.09-1.28)	0.07 (0.04-0.10)
	Prevalence of low TTR*	few low TTR	5	0.86 (0.77-0.96)	1.19 (1.05-1.36)	0.08 (0.02-0.13)
		many low TTR	5	0.84 (0.76-0.93)	1.39 (1.14-1.69)	0.10 (0.06-0.15)
	Categorised mean TTR*	TTR high	5	0.84 (0.76-0.92)	1.24 (1.08-1.42)	0.09 (0.04-0.15)
		TTR low	5	0.87 (0.81-0.94)	1.34 (1.09-1.65)	0.09 (0.05-0.13)
	Categorised sd TTR*	SD high	5	0.84 (0.73-0.96)	1.29 (1.08-1.54)	0.10 (0.04-0.16)
		SD low	5	0.86 (0.80-0.93)	1.27 (1.08-1.49)	0.08 (0.04-0.13)
	Quality of anticoagulation**	TTR high, SD high	1	0.77 (0.70-0.86)	1.44 (1.24-1.68)	0.15 (0.09-0.21)
		TTR high, SD low	4	0.86 (0.76-0.97)	1.17 (1.04-1.31)	0.07 (0.01-0.13)
		TTR low, SD high	4	0.88 (0.79-0.98)	1.21 (1.02-1.42)	0.07 (0.02-0.12)
		TTR low, SD low	1	0.86 (0.80-0.93)	1.64 (1.23-2.19)	0.12 (0.06-0.18)

LR-, LR+: negative and positive likelihood ratio, respectively; PSEP: power of separation.

AF: atrial fibrillation; N: number of studies; PINRR: proportion of INR's in range; TTR: time within therapeutic range; VTE: venous thrombo-embolism.

* Categorisation of prevalence of low TTR, mean TTR and standard deviation TTR based on upper and lower half.

** Quality of anticoagulation is a combination of categories from mean TTR and sd TTR.

SAMe-TT_2R_2	Sensitivity analysis		N	LR-	LR+	PSEP
≥ 3	Full analysis		10	0.96 (0.91-1.02)	1.24 (1.09-1.40)	0.06 (0.02-0.10)
	Indication	AF	9	0.96 (0.90-1.02)	1.24 (1.09-1.41)	0.06 (0.02-0.10)
		VTE	1	0.98 (0.95-1.02)	1.23 (0.85-1.80)	0.05 (-0.04-0.14)
	TTR method	PINRR	1	0.96 (0.93-0.99)	1.95 (0.99-3.84)	0.12 (0.02-0.22)
		Rosendaal	9	0.96 (0.91-1.02)	1.19 (1.06-1.34)	0.06 (0.02-0.09)
	Origin	original data	2	1.04 (0.82-1.32)	1.12 (0.88-1.42)	0.03 (-0.05-0.12)
		simulation	8	0.91 (0.87-0.96)	1.33 (1.14-1.55)	0.08 (0.05-0.11)
	Prevalence of low TTR*	few low TTR	5	0.89 (0.83-0.94)	1.29 (1.12-1.48)	0.09 (0.05-0.13)
		many low TTR	4	1.06 (0.91-1.24)	1.23 (1.00-1.50)	0.04 (-0.03-0.11)
	Categorised mean TTR*	TTR high	4	0.93 (0.90-0.97)	1.34 (1.15-1.57)	0.09 (0.04-0.13)
		TTR low	5	0.99 (0.85-1.15)	1.20 (1.01-1.43)	0.05 (-0.01-0.11)
	Categorised sd TTR*	SD high	4	0.99 (0.84-1.17)	1.14 (0.96-1.34)	0.04 (-0.02-0.11)
		SD low	5	0.93 (0.86-1.00)	1.45 (1.13-1.85)	0.09 (0.05-0.13)
	Quality of anticoagulation**	TTR high, SD high	1	0.96 (0.92-1.01)	1.31 (0.93-1.84)	0.07 (-0.02-0.17)
		TTR high, SD low	3	0.92 (0.88-0.96)	1.36 (1.15-1.61)	0.09 (0.04-0.14)
		TTR low, SD high	3	1.00 (0.77-1.31)	1.09 (0.92-1.30)	0.04 (-0.04-0.11)
		TTR low, SD low	2	0.95 (0.79-1.14)	1.61 (0.76-3.43)	0.09 (-0.00-0.18)

LR-, LR+: negative and positive likelihood ratio, respectively; PSEP: power of separation.

AF: atrial fibrillation; N: number of studies; PINRR: proportion of INR's in range; TTR: time within therapeutic range; VTE: venous thrombo-embolism.

* Categorisation of prevalence of low TTR, mean TTR and standard deviation TTR based on upper and lower half.

** Quality of anticoagulation is a combination of categories from mean TTR and sd TTR.
