# Supplementary material for: Clinical usefulness of the SAMe-TT2R2 score: A systematic review and simulation meta-analysis
Source: PLoS One. 2018 Mar 13;13(3):e0194208. doi: 10.1371/journal.pone.0194208 (PMC5849337; doi:10.1371/journal.pone.0194208)
Supplement: S1 Fig — Shows the difference between the simulated and original values. (PDF) [file pone.0194208.s005.pdf]

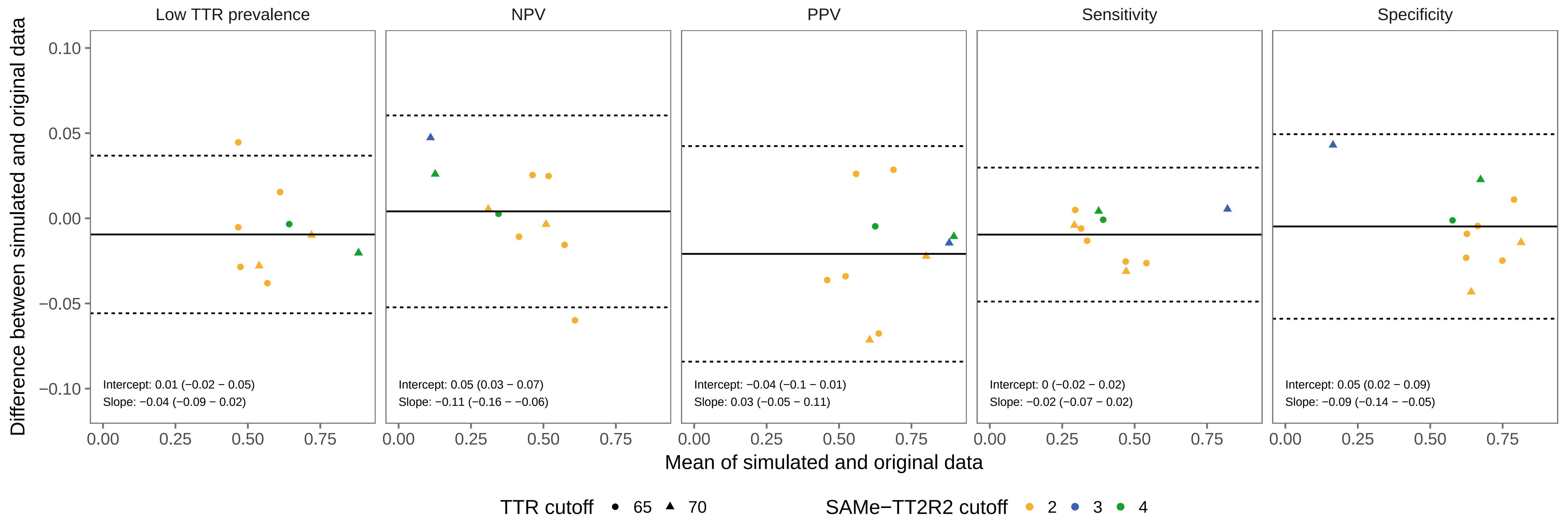
Low TTR prevalence
NPV
PPV
Sensitivity
Specificity
Difference between simulated and original data
0.10
0.05
0.00
−0.05
−0.10
Intercept: 0.01 (−0.02 − 0.05)
Slope: −0.04 (−0.09 − 0.02)
Intercept: 0.05 (0.03 − 0.07)
Slope: −0.11 (−0.16 − −0.06)
Intercept: −0.04 (−0.1 − 0.01)
Slope: 0.03 (−0.05 − 0.11)
Intercept: 0 (−0.02 − 0.02)
Slope: −0.02 (−0.07 − 0.02)
Intercept: 0.05 (0.02 − 0.09)
Slope: −0.09 (−0.14 − −0.05)
0.00 0.25 0.50 0.75
Mean of simulated and original data
TTR cutoff 65 70
SAMe−TT2R2 cutoff 2 3 4
