# Supplementary material for: Clinical usefulness of the SAMe-TT2R2 score: A systematic review and simulation meta-analysis
Source: PLoS One. 2018 Mar 13;13(3):e0194208. doi: 10.1371/journal.pone.0194208 (PMC5849337; doi:10.1371/journal.pone.0194208)
Supplement: S2 Fig — Uses cutoffs of ≥2 and ≥3 to predict a TTR <70%. PSEP: power of separation; TTR: time in therapeutic range. (PDF) [file pone.0194208.s006.pdf]

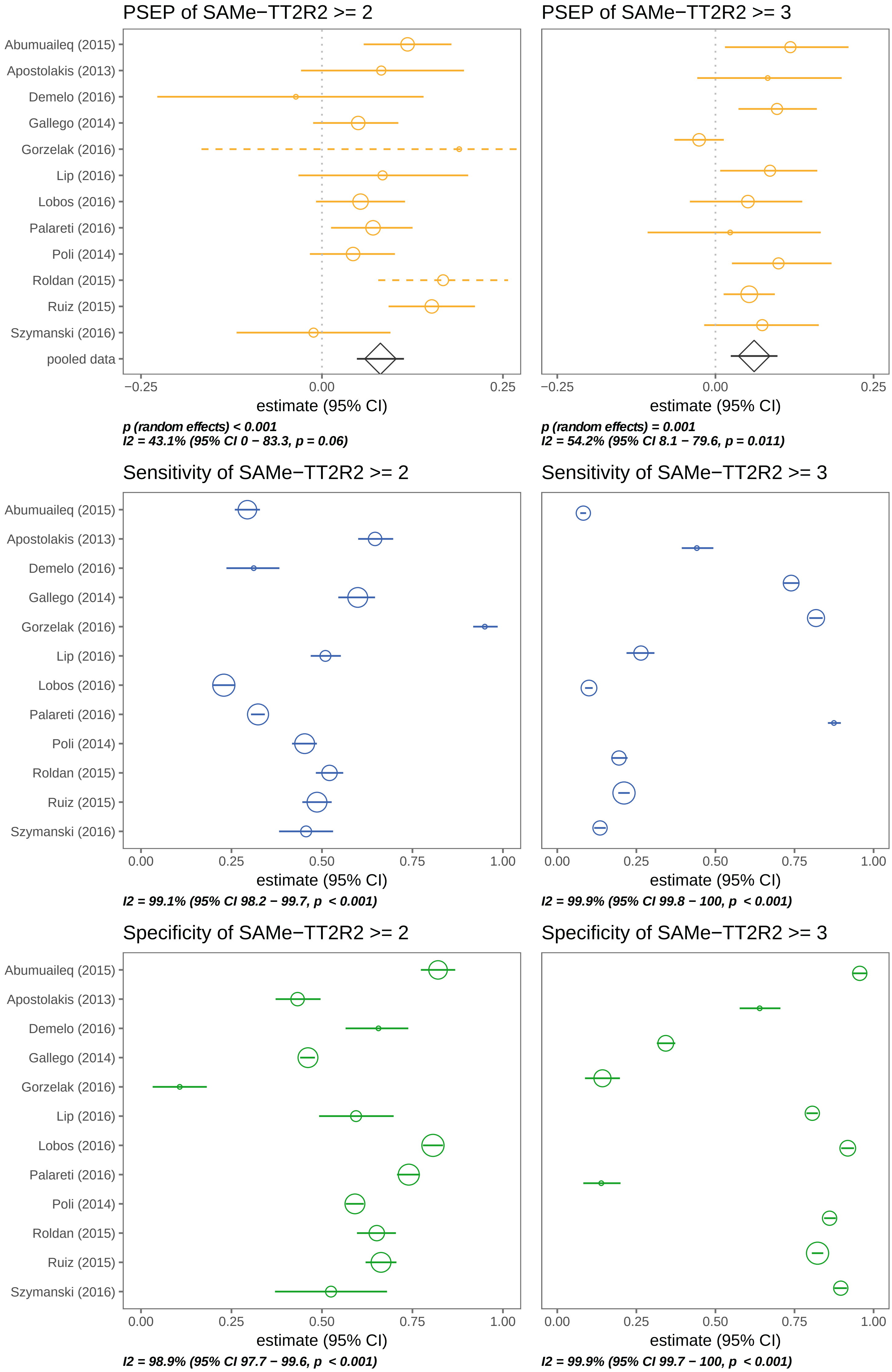

PSEP of SAMe−TT2R2 >= 2
PSEP of SAMe−TT2R2 >= 3
Abumuaileq (2015)
Apostolakis (2013)
Demelo (2016)
Gallego (2014)
Gorzelak (2016)
Lip (2016)
Lobos (2016)
Palareti (2016)
Poli (2014)
Roldan (2015)
Ruiz (2015)
Szymanski (2016)
pooled data
−0.25
0.00
0.25
estimate (95% CI)
p (random effects) < 0.001
I2 = 43.1% (95% CI 0 − 83.3, p = 0.06)
p (random effects) = 0.001
I2 = 54.2% (95% CI 8.1 − 79.6, p = 0.011)
Sensitivity of SAMe−TT2R2 >= 2
Sensitivity of SAMe−TT2R2 >= 3
0.00
0.25
0.50
0.75
1.00
I2 = 99.1% (95% CI 98.2 − 99.7, p < 0.001)
I2 = 99.9% (95% CI 99.8 − 100, p < 0.001)
Specificity of SAMe−TT2R2 >= 2
Specificity of SAMe−TT2R2 >= 3
I2 = 98.9% (95% CI 97.7 − 99.6, p < 0.001)
I2 = 99.9% (95% CI 99.7 − 100, p < 0.001)
