# Supplementary figures and images for: Clinical usefulness of the SAMe-TT2R2 score: A systematic review and simulation meta-analysis
Source: PLoS One. 2018 Mar 13;13(3):e0194208. doi: 10.1371/journal.pone.0194208 (PMC5849337; doi:10.1371/journal.pone.0194208)

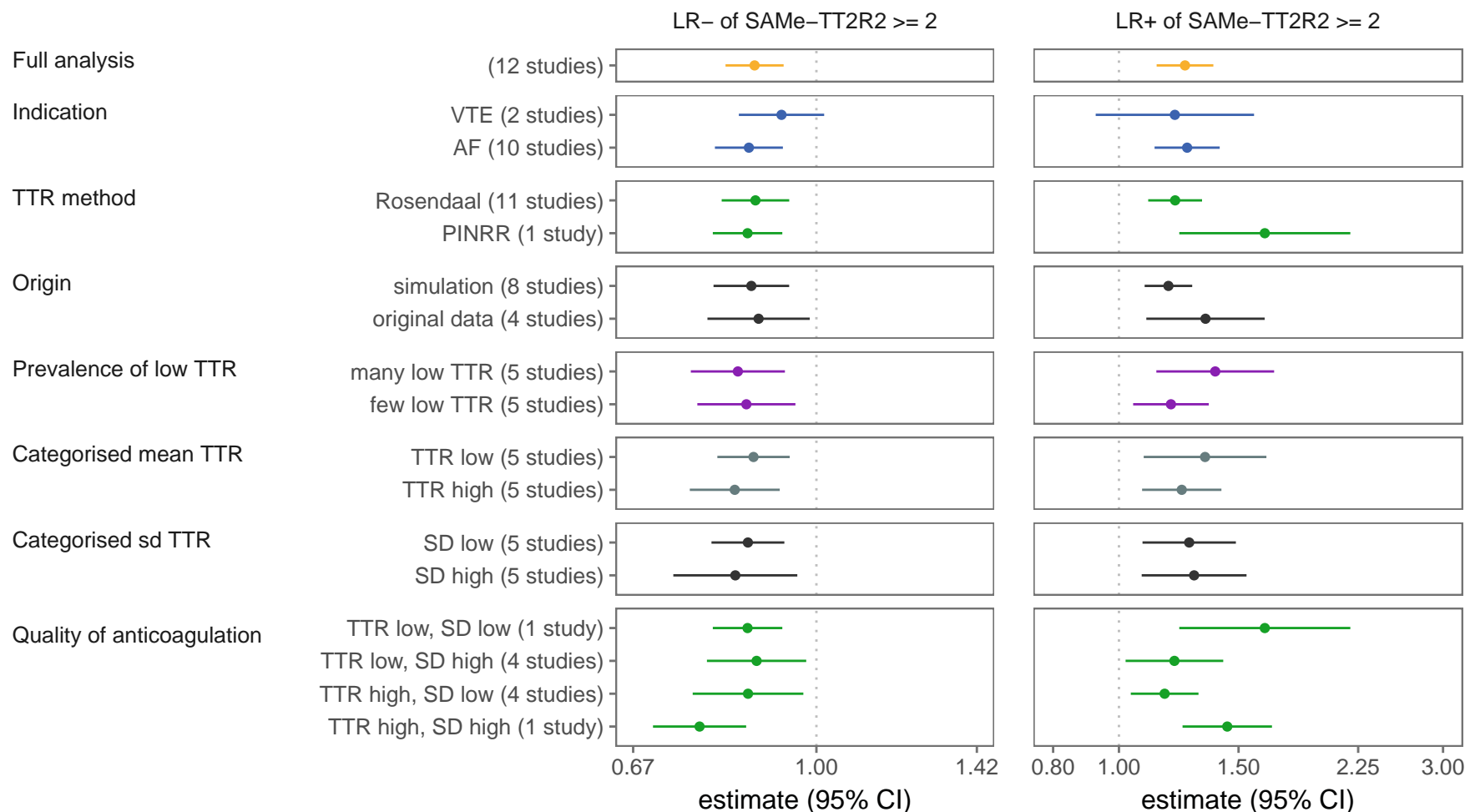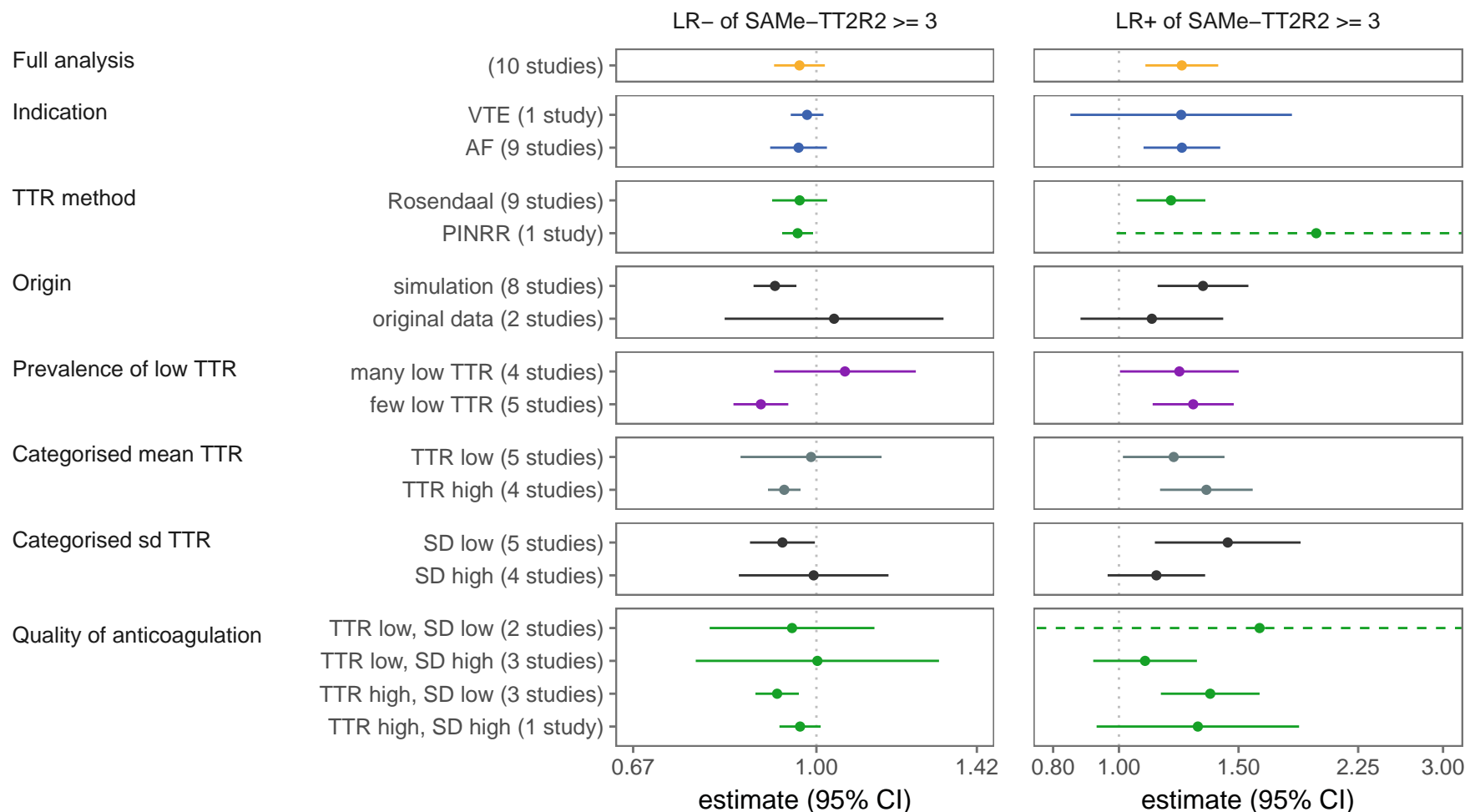

Supplement: S3 Fig — Shows how different indications or methods of TTR measurement change the results. Dotted lines represent confidence intervals that were too wide to be displayed properly. (PDF) [file pone.0194208.s007.pdf]
